# Genome-wide analysis of wheat calcium ATPases and potential role of selected *ACA*s and *ECA*s in calcium stress

**DOI:** 10.1186/s12870-017-1112-5

**Published:** 2017-10-27

**Authors:** Roohi Aslam, Lorraine E. Williams, Muhammad Faraz Bhatti, Nasar Virk

**Affiliations:** 1 Atta-ur-Rahman School of Applied Biosciences, National University of Sciences and Technology, Islamabad, 44000 Pakistan; 2 Biological Sciences, University of Southampton, Southampton, SO17 1BJ UK

**Keywords:** Calcium, P_2_- type, *ACA*s, *ECA*s, qRT-PCR

## Abstract

**Background:**

P_2_- type calcium ATPases (*ACA*s-auto inhibited calcium ATPases and *ECA*s-endoplasmic reticulum calcium ATPases) belong to the P- type ATPase family of active membrane transporters and are significantly involved in maintaining accurate levels of Ca^2+^, Mn^2+^ and Zn^2+^ in the cytosol as well as playing a very important role in stress signaling, stomatal opening and closing and pollen tube growth. Here we report the identification and possible role of some of these ATPases from wheat.

**Results:**

In this study, *ACA* and *ECA* sequences of six species (belonging to Poaceae) were retrieved from different databases and a phylogenetic tree was constructed. A high degree of evolutionary relatedness was observed among P_2_ sequences characterized in this study. Members of the respective groups from different plant species were observed to fall under the same clade. This pattern highlights the common ancestry of P_2−_ type calcium ATPases. Furthermore, qRT-PCR was used to analyse the expression of selected *ACAs* and *ECAs* from *Triticum aestivum* (wheat) under calcium toxicity and calcium deficiency. The data indicated that expression of *ECA*s is enhanced under calcium stress, suggesting possible roles of these ATPases in calcium homeostasis in wheat. Similarly, the expression of *ACA*s was significantly different in plants grown under calcium stress as compared to plants grown under control conditions. This gives clues to the role of *ACA*s in signal transduction during calcium stress in wheat.

**Conclusion:**

Here we concluded that wheat genome consists of nine P_2B_ and three P_2A_ -type calcium ATPases. Moreover, gene loss events in wheat ancestors lead to the loss of a particular homoeolog of a gene in wheat. To elaborate the role of these wheat ATPases, qRT-PCR was performed. The results indicated that when plants are exposed to calcium stress, both P_2A_ and P_2B_ gene expression get enhanced. This further gives clues about the possible role of these ATPases in wheat in calcium management. These findings can be useful in future for genetic manipulations as well as in wheat genome annotation process.

## background

Calcium is one of the most important elements required to perform a variety of functions in plants. Various membrane proteins are responsible for maintaining an accurate level of calcium within the plant. Among them, P_2_- type ATPases have significant importance. The P_2_- type ATPases are generally recognized by the formation of a phosphorylated intermediate (hence called P- type), by being inhibited by vanadate and by having a large number of common sequence motifs [1, 2]. The presence of 8–12 transmembrane segments and N and C termini exposed to the cytoplasm is characteristic of P- type ATPases [3]. Subcellular localization of P_2_- type Ca- ATPases generally include cell membrane [4, 5] or endoplasmic reticulum [6] and Golgi [2, 7].

The P_2_- type ATPases are further divided into P_2A_ and P_2B_- types [8]. P_2A_- type ATPases form a distinct set of ER-type Ca^2+^ ATPases, generally called *ECA*s and are closely related to the animal sarco-endoplasmic reticulum Ca^2+^pump SERCA1 [9]. The P_2B_- type ATPases are characterized by the binding of calmodulin to their auto inhibitory terminal domains and show similarities to animal CaM-stimulated Ca^2+^ ATPases (PMCA). They are generally known as *ACA*s [1, 9]. In lower plants such as *P. patens* and higher plants such as *A. thaliana*, the calmodulin-binding domains (CMBDs) of *ACA*s are located in the N-terminus [10]. However, no N-terminus CMBD could be identified in P_2B_- type ATPases from the chlorophytes *O. tauri* and *C. reinhardtii* [3].

Calcium ATPases are considered equally important both in plants as well as animals, because of their significant roles in both clades of life. For instance, the proper development and functioning of osteoclasts require a sophisticated control by PMCAs over intra and extracellular concentrations of calcium ions [11, 12]. An increase in expression level of plasma membrane calcium ATPase (PMCA) isoforms 1 and 4 occur during a late phase of osteoclast differentiation [13, 14]. However, less expression of these isoforms results in low bone mass in mice which indicated a clear role of PMCAs in the proper development of osteoclast and bone homeostasis [15] These ATPases also have significant importance in plants. For example, *ACA8* and one its closest homolog is generally required for limiting the growth of bacteria. *ACA8* is also required for proper plant development [16]. Another study indicated that *ACA2* plays a role against osmotic stress in plants. The evidence comes from the fact that a yeast mutant (K616) which is deficient in calcium pump can grow under salinity stress after heterologous expression of endoplasmic reticulum located *Arabidopsis thaliana* calcium ATPase *ACA2* in it [17]. The *ACA4* is situated in vacuolar membranes and provide resistance against osmotic stress (such as NaCl, KCl, and mannitol) as observed through various experiments performed using yeast models [9].

Monocotyledons refer to a group of flowering plants whose seed contain only one embryonic leaf or cotyledon. The stem is usually unbranched and fleshy whereas, their roots are short and stringy. Monocots are quite diverse and comprise one-quarter of all flowering plants on earth (about 60,000 species). Orchidaceae is the largest monocotyledon plant family which includes more than 20,000 species. Another important monocot family is Poaceae (also known as the grass family) which includes a large number of economically important cereals such as rice, wheat, maize etc. Cereals constitute a most prevalent group of crops across the world whose cultivation exceeds 20% of the global land area [18]. According to “Crop Prospects and Food Situation Report” FAO estimates that world cereal production will reach around 2500 million hectares in the coming years which show a tremendous increase. Interestingly, among cereals, wheat occupies the first position in terms of production and it accounts for a total of 20% of the calories consumed by human beings [19]. United Nations estimates that by 2050 the world’s population will be 9.1 billion and 70% of the world’s population will become urban [20]. In order to feed such a large urban living population net, wheat production must increase by 70% [20]. Therefore, attempts should be made to engineer wheat plants which may have the ability to grow at a fast rate with increased grain yield. Also, these plants should be able to withstand harsh environmental conditions. Only then it will be possible to cope with the demand of increase, food supply in the world.

Modern bread wheat originated as a result of two independent hybridization events in nature. The first hybridization event occurred between *Triticum urartu* (2n = 2× = 14, genome AA) and *Aegilops speltoides* (2n = 2× = 14, genome BB) 300,000–500,000 BP, which led to the production of tetraploid wild emmer wheat (AABB, *Triticum dicoccoides*). Early agrarians planted the seeds of tetraploid wild emmer (AABB). Domesticated emmer spread across the entire Asia, Europe and Africa [21]. This spread of cultivation brought it closer to another species *Aegilops tauschii* (the donor of the DD genome) in the Caspian basin where hybridization is presumed to have taken place (about 8000 years ago), giving rise to hexaploid wheat [20]. From those beginnings, the cultivation of hexaploid wheat (bread wheat or *Triticum aestivum*) has spread to the far reaches of the globe. Due to having a hexaploid genome, wheat is a polyploid organism. More specifically, modern bread wheat is an allohexaploid having 21 pairs of chromosomes, which are composed of 7 homoeolog groups (A1, B1, D1...A7, B7, D7). Wheat genome has been sequenced recently and a comprehensive genome wide analysis of the wheat genome was released in 2012 [22]. This information was used to create assemblies of wheat genes in an orthologous gene family framework. The subsequent data is available in URGI [23] and PGSB [24]. Most recently The Universal Protein Resource Knowledgebase (UniProtKB) [25] and Ensembl Plants [26] has also annotated some of the wheat proteins. Recent advances in the field of bioinformatics and the availability of many sequenced genomes (of grasses) greatly facilitates the investigation of the evolutionary history and diversity of P_2_- type ATPases among grasses. In this study, genome wide analysis of wheat genome was done to predict the possible wheat calcium ATPases. Phylogenetic analysis was also conducted to find out the evolutionary relationship among different members of the family Poaceae. Furthermore, the effect of calcium stress (deficiency and excess) on P_2_ - type ATPases expression was also demonstrated using the qRT-PCR technique.

## Methods

### Phylogenetic analysis

In order to conduct the phylogenetic analysis, sequences of *ACA*s and *ECA*s from different grasses were retrieved from different databases (Table 1). The sequences chosen were believed to span the confirmed *ACA*s and *ECA*s genes across the plant kingdom. *Oryza sativa* annotated *ACA*s and *ECA*s sequences were retrieved from Michigan State University Rice Genome Annotation Project (MSU) [27] and were cross verified with rice calcium ATPase sequences given in membrane transporter database ARAMEMNON [28] and Rice Annotation Project (RAP) [29]. *Oryza sativa* calcium ATPases sequences were used to do BLAST searches in UniProtKB [25] and Ensembl Plants [26] databases to retrieve calcium ATPase sequences of different grasses. A list of the databases used along with the species name is given in Table 1. Full length protein sequences were used in the final tree. However, partial length sequences were used if full length sequences were not available. Length of sequences was determined on the basis of corresponding *Oryza sativa* calcium ATPase sequence. Sequences of six monocot species (*Triticum urartu*, *Triticum aestivum*, *Oryza sativa*, *Oryza brachyantha*, *Oryza barthii* and *Sorghum bicolor*) were used in the construction of the tree.

**Table 1:** List of different plant species along with accession numbers

Taxon	Accession numbers	Sequence length	Databases
*ACA1*			
*Triticum aestivum*	TRIAE_CS42_4AS_TGACv1_306881_AA1014450.1	1020	Ensembl Plants
*Triticum aestivum*	TRIAE_CS42_4BL_TGACv1_322716_AA1072800.2	1020	Ensembl Plants
*Triticum aestivum*	TRIAE_CS42_4DL_TGACv1_342814_AA1122680.1	1020	Ensembl Plants
*Triticum urartu*	M7ZNL4	1020	UniProtKB
*Brachypodium distachyon*	Bradi1g70920.1	1020	ARAMEMNON
*Oryza sativa*	LOC_Os03g10640	1019	MSU
*Sorghum bicolor*	C5WTS5	1020	UniProtKB
*Oryza brachyantha*	J3ll50	1031	UniProtKB
*ACA2*			
*Triticum aestivum 5AS*	TRIAE_CS42_5AS_TGACv1_393493_AA1273190.4	1020	Ensembl Plants
*Triticum aestivum 5BS*	TRIAE_CS42_5BS_TGACv1_423347_AA1374870.1	1020	Ensembl Plants
*Triticum aestivum 5DS*	TRIAE_CS42_5DS_TGACv1_458228_AA1492790.1	1020	Ensembl Plants
*Triticum urartu*	M8A7X8	946 *	UniProtKB
*Brachypodium distachyon*	Bradi4g03130.1	1019	ARAMEMNON
*Oryza sativa*	LOC_Os12g39660.1	1020	MSU
*Oryza barthii*	A0A0D3HW73	1020	UniProtKB
*ACA3*			
*Triticum aestivum*	TRIAE_CS42_4AL_TGACv1_288269_AA0942920.1	1052	Ensembl Plants
*Triticum aestivum*	TRIAE_CS42_U_TGACv1_641388_AA2093540.1	1052	Ensembl Plants
*Triticum aestivum*	TRIAE_CS42_4DS_TGACv1_361699_AA1171710.1	1050	Ensembl Plants
*Triticum Urartu*	M8AJX4	1536	UniProtKB
*Brachypodium distachyon*	Bradi1g14630.1	1020	ARAMEMNON
*Oryza sativa*	LOC_Os03g42020.1	1033	MSU
*Sorghum bicolor*	C5WSB3	1033	UniProtKB
*vOryza brachyantha*	J3LQU0	986*	UniProtKB
*Oryza barthii*	A0A0D3FLA5	1033	UniProtKB
*ACA4*			
*Triticum Urartu*	M7ZET5	998*	UniProtKB
*Brachypodium distachyon*	Bradi4g43300.1	1035	ARAMEMNON
*Oryza sativa*	LOC_Os11g04460.1	1017	MSU
*Sorghum bicolor*	C5Y458	1037	UniProtKB
*Oryza barthii*	A0A0D3HR67	1039	UniProtKB
*ACA7*			
*Triticum aestivum*	TRIAE_CS42_1BL_TGACv1_030749_AA0099780.1	1042	Ensembl Plants
*Triticum aestivum*	TRIAE_CS42_1AL_TGACv1_001355_AA0029220.1	980*	Ensembl Plants
*Triticum aestivum*	TRIAE_CS42_1DL_TGACv1_062322_AA0212540.1	980*	Ensembl Plants
*Triticum Urartu*	M7YR54	992*	UniprotKB
*Brachypodium distachyon*	Bradi2g21180.1	1041	ARAMEMNON
*Oryza sativa*	LOC_Os05g41580.1	1057	MSU
*Sorghum bicolor*	C5Z0B0	1042	UniProtKB
*Oryza brachyantha*	J3M8H2	1038	UniProtKB
*Oryza barthii*	A0A0D3G9C7	1073	UniProtKB
*ACA8*			
*Triticum aestivum*	TRIAE_CS42_1BL_TGACv1_031294_AA0110960.1	1020	Ensembl Plants
*Triticum aestivum*	TRIAE_CS42_1AL_TGACv1_001862_AA0035990.1	1024	Ensembl Plants
*Triticum aestivum*	TRIAE_CS42_1DL_TGACv1_061321_AA0192370.1	1034	Ensembl Plants
*Brachypodium distachyon*	Bradi3g26890.1	1025	ARAMEMNON
*Oryza sativa*	LOC_Os10g28240.1	1035	MSU
*Sorghum bicolor*	C5X1K4	1012	C5X1K4
*Oryza brachyantha*	J3N2P8	1049	UniProtKB
*Oryza barthii*	A0A0D3HDQ0	1032	A0A0D3HDQ0
*Unidentified*			
*Triticum aestivum*	TRIAE_CS42_7DS_TGACv1_621790_AA2026140.1	1083	Ensembl Plants
*Triticum aestivum*	TRIAE_CS42_U_TGACv1_641800_AA2104440.1	1083	Ensembl Plants
*Triticum aestivum*	TRIAE_CS42_U_TGACv1_641800_AA2104450.3	1082	Ensembl Plants
*Triticum Urartu*	M7YGM5	1050	UniProtKB
*Brachypodium distachyon*	Bradi3g40640.1	1094	ARAMEMNON
*Sorghum bicolor*	C5YI87	1087	UniProtKB
*Oryza brachyantha*	J3MUF6	1086	UniProtKB
*Oryza barthii*	A0A0D3H254	1016	UniProtKB
*ACA11*			
*Triticum aestivum*	TRIAE_CS42_2BL_TGACv1_129973_AA0400750.3	1087	Ensembl Plants
*Triticum aestivum*	TRIAE_CS42_2AL_TGACv1_093051_AA0270470.1	1081	Ensembl Plants
*Triticum aestivum*	TRIAE_CS42_2DL_TGACv1_159040_AA0531140.1	1228	Ensembl Plants
*Brachypodium distachyon*	Bradi5g20890.1	1082	ARAMEMNON
*Oryza sativa*	LOC_Os04g51610.1	1089	MSU
*Sorghum bicolor*	C5YFI8	1092	UniProtKB
*Oryza brachyantha*	J3 M160	1084	UniProtKB
*Oryza barthii*	A0A0D3FZV8	1013	UniProtKB
*Unidentified*			
*Triticum aestivum*	TRIAE_CS42_6AS_TGACv1_485501_AA1546480.1	1094	Ensembl Plants
*Triticum aestivum*	TRIAE_CS42_6BS_TGACv1_514490_AA1660470.1	1097	Ensembl Plants
*Triticum aestivum*	TRIAE_CS42_6DS_TGACv1_542558_AA1724300.1	1097	Ensembl Plants
*Triticum Urartu*	M7ZL44	1130	UniProtKB
*Brachypodium distachyon*	Bradi3g05697.1	1027	ARAMEMNON
*Oryza brachyantha*	J3LA39	1088	UniProtKB
*Oryza barthii*	A0A0D3F1F8	1084	UniProtKB
*ACA6*			
*Triticum aestivum*	TRIAE_CS42_3AL_TGACv1_194974_AA0643030.1	1043	Ensembl Plants
*Triticum aestivum*	TRIAE_CS42_3B_TGACv1_225697_AA0811210.1	1043	Ensembl Plants
*Triticum aestivum*	TRIAE_CS42_3DL_TGACv1_251172_AA0878350.1	1043	Ensembl Plants
*Brachypodium distachyon*	Bradi2g60324.1	1051	ARAMEMNON
*Oryza sativa*	loc os01g71240	1043	MSU
*Oryza brachyantha*	J3L7P9	1043	UniProtKB
*ECA1*			
*Triticum aestivum 4DL*	TRIAE_CS42_4BL_TGACv1_322129_AA1068800.1	1105	Ensembl Plants
*Triticum aestivum 4BL*	TRIAE_CS42_4AS_TGACv1_306876_AA1014390.1	1068	Ensembl Plants
*Triticumaestivum 4AS_V2*	TRIAE_CS42_4DL_TGACv1_342374_AA1111770.2	873*	Ensembl Plants
*Brachypodium distachyon*	I1H6T2	1062	ARAMEMNON
*Oryza sativa*	Q8H8w1	845*	MSU
*Sorghum bicolor*	C5WP97	1061	UniProtKB
*Oryza barthii*	A0A0D3FGZ7	1058	UniProtKB
*ECA3*			
*Triticum aestivum 4DS*	IWGSC_chr4DS_ab_k71	977*	URGI
*Triticum aestivum 4BS*	IWGSC_chr4BS_ab_k71	1002	URGI
*Triticum aestivum 4A*	N/A	N/A	N/A
*Brachypodium distachyon*	Bradi1g09810.1	1002	UniProtKB
*Oryza sativa*	LOC_Os03g52090.1	1217	MSU
*Sorghum bicolor*	A0A1B6QIC1	1000	UniProtKB
*Oryza brachyantha*	J3LSI2	1000	UniProtKB
*Oryza barthii*	A0A0D3FNM9	1078	UniProtKB
*ECA2*			
*Triticum aestivum*	TRIAE_CS42_1BS_TGACv1_049567_AA0157010.1;	1057	UniProtKB
*Triticum aestivum*	TRIAE_CS42_1AS_TGACv1_020544_AA0078240.1	1057	Ensembl Plants
*Triticum aestivum*	TRIAE_CS42_1DS_TGACv1_080510_AA0249290.1	1054	Ensembl Plants
*Brachypodium distachyon*	I1HME9	1038	UniProtKB
*Triticum urartu*	M8AS38	848*	UniProtKB
*Sorghum bicolor*	C5YYZ2	1058	UniProtKB
*Oryza brachyantha*	J3M3F0	1057	UniProtKB

* Partial sequences

The amino acid sequence alignment was performed using CLUSTAL W. The Gap open penalty was 10 whereas, the gap extension penalty was 0.1. To perform Evolutionary analysis MEGA version 7 was used [30] and a phylogenetic tree was constructed using Maximum Likelihood method based on the JTT matrix-based model [31]. A matrix of pairwise distances was estimated using a JTT model. Neighbor-Join and BioNJ algorithms were applied to this matrix to get an initial tree(s) for the heuristic search. Topology was then selected with superior log likelihood value. The tree is drawn to scale, with branch lengths measured in the number of substitutions per site. All positions containing gaps and missing data were eliminated from the dataset.

### Growth of wheat plants

*Triticum aestivum* (Var. Sehar-06) plants were grown under calcium stress using a hydroponic system. Prior to germination seeds were surface sterilized using 1% bleach solution and were left for germination in the dark for five days. Seeds were grown for 14 days on standard media, according to Lombnaes and Singh [32]. The fourteenth day of growth on standard Lombnaes media is referred to as D0 in this paper. In D0, standard Lombnaes media was modified to induce deficiency and toxicity stress. In order to induce calcium deficiency, no calcium was added to the standard Lombnaes media. For the induction of calcium toxicity, 8 mM of calcium was added to the standard Lombnaes media. Normal 2 mM calcium concentration was maintained for control plants. Prior to transfer to calcium deficiency and toxic medium roots of plants were washed with ddH_2_0 thrice. The plants were grown for a further 21 days. Nine plants (three for each set) were harvested on days 7, 14, and 21. Fresh weight (FW) of roots and shoots was noted after harvesting the plants. The roots and shoots were snap frozen prior to preservation at −80 °C. The significant difference between fresh weight values was determined using Student’s t-test.

The plants were cultivated in an environmentally controlled growth room with the temperature set at 21 °C/16 °C (day/night), humidity maintained at 55–65%. The photoperiod was kept for 16 h. at a quantum flux density (PAR) of 220 μmol m^−2^ s^−^.

### RNA extraction and cDNA synthesis

For RNA extraction (of *Triticum aestivum*) roots and shoots were finely ground using liquid nitrogen. Finely ground wheat tissue (0.5 ml) was put into Eppendorf tube to which 1 ml TRIzol (Invitrogen, CA, USA) reagent was added. Eppendorf was vortexed vigorously before the addition of Chloroform 25% (*v*/v). The mixture was left for incubation at room temperature for five minutes, followed by centrifugation at 12,000 *g* at 4 °C for 15 min. After centrifugation was completed, the colorless upper phase was transferred to a new tube and 50% (*v*/v) isopropyl alcohol was added. The mixture was vortexed briefly and was left at room temperature for 10 min. The mixture was centrifuged at 12,000 *g* at 4 °C to obtain RNA pellet. The pellet was re suspended in 1 ml 75% (v/v) ethanol and vortexed briefly and was centrifuged at 7600 *g* at 4 °C for 5 min. This step was repeated three times. The pellet was left to air dry at room temperature for at least 5–10 min after removal of supernatant. The pellet was suspended in freshly prepared 30 μL TE buffer (pH 7.0). RNA samples were treated with DNase to prevent any possible genomic contamination. Extracted RNA was used to synthesize first strand complementary DNA (cDNA) using cDNA synthesis kit (Invitrogen), following manufacturer’s instructions.

### qRT- PCR

Real time PCR was performed to corroborate the expression of selected ATPases under calcium stress. The primers were designed and validated using the BLAST tool of NCBI whereas, primer sequence for “actin” was obtained from a previously published work [33]. Dissociation curve for each reaction was analysed to determine primer specificity. All the primers used in this study are listed in Table 2. Real-time PCR reaction was performed using the SYBR Green Kit (Invitrogen). To perform the reaction, 2.5 ng of template DNA, 0.3 μM of forward and reverse primers, 1X SYBR-green master mix and sterile 18 Ω H_2_O up to 20 μL was used in a 96 well plate format. The reaction was run on an Opticon DNA Engine Continuous Fluorescence Detector (Applied Biosystems 7000 Real-time PCR system). The conditions used were 95 °C for 2 min before cycling forty times at 95 °C for 50 s, 60 °C for 50s, 70 °C for 5 min and a final extension time of 71 °C for 10 min. The house keeping gene “actin” was used for normalization of cDNA variance among the samples. Relative expression values were calculated following the method described by Pfaffl [34].

**Table 2:** List of qRT-PCR primers

Primer pairs	Primers	Sequence (5′ - 3′)
1	TaECA1-F	CAGTTTCAATGAATGGCTTTTGGTC
	TaECA1-R	CTTTCTGGCCCGAGCTGTCA
2	TaECA3-F	TCTCTACTTGTCATTCACCCATGG
	TaECA3-R	ATGGAGACACTGAGAAAAGAGCT
3	TaACA2-F	CGTCTTCTGCCAGGTGTTCA
	TaACA2-R	GCCGAGGAATTGGACCATGA
4	TaACA3-F	AGGGCATGTTGGAGAACTCT
	TaACA3-R	GCCAAAGAGGATGCAGACGA
5	TaACA4-F	GCTGGCAATTCTGGTTGGTG
	TaACA4-R	TATGTCATCAGGGCCGTTGG
6	Actin-F	ACCTTCAGTTGCCCAGCAAT
	Actin-R	CAGAGTCGAGCACAATACCAGTTG

## Results

### Sequence retrieval and phylogenetic analysis

**Fig. 1:**
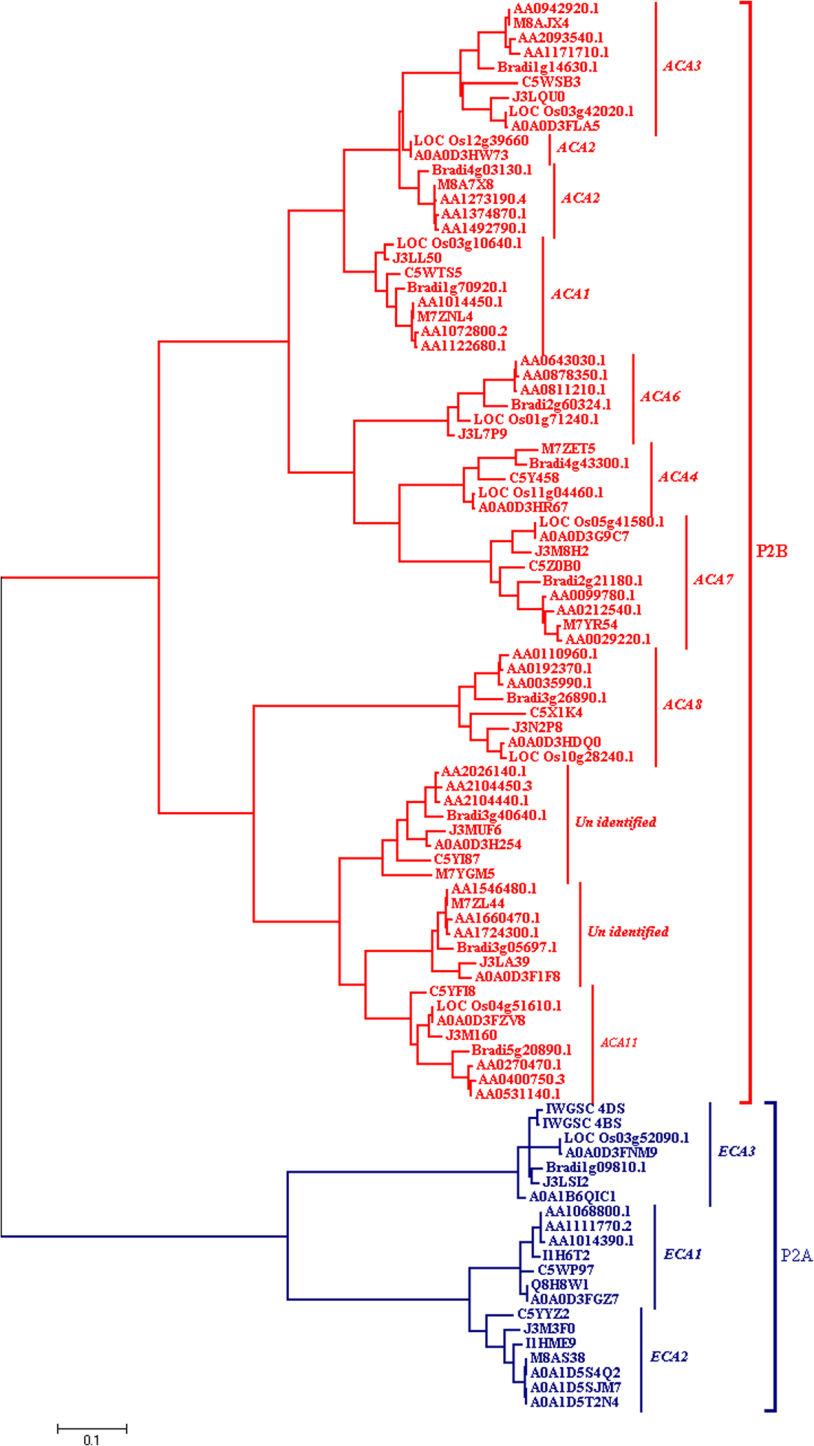
Molecular Phylogenetic analysis by Maximum Likelihood method. Tree with highest log likelihood is shown in the figure. For tree construction, the positions containing gaps were eliminated. There were a total of 372 positions in the final dataset

To determine the evolutionary relatedness among P_2_- type calcium ATPases from *Triticum aestivum*, *Triticum urartu*, *Brachypodium distachyon*, *Oryza sativa*, *Sorghum bicolor*, *Oryza brachyantha* and *Oryza barthii,* a phylogenetic tree was constructed (Fig. 1). Ninety six amino acid sequences were used in the construction of phylogenetic tree using Maximum Likelihood method (Fig. 1). Phylogenetic analysis revealed that P_2_- type calcium ATPases formed two distinct groups referred as P_2A_ and P_2B_. Overall, P_2_ sequences used in this study displayed a high degree of evolutionary relatedness. The investigation further revealed that each species had members of the respective as P_2A_ and P_2B_ groups and those in each group showed a high degree of similarity. This pattern highlights the common ancestry of P_2_- type calcium ATPases in distinct species. Furthermore, nine P_2B_- type and three P_2A_- type calcium ATPases have been identified in wheat.

Wheat is hexaploid so three homoeologs (A, B and D) for each gene are expected [35]. For all wheat, calcium ATPases studied in this work, three homoeologs were found except for *ECA3*. Two homoeologs i.e., 4BS and 4DS were found for this gene in the databases searched, whereas, the third one “A” was not found. Also, no *ECA3* sequence was found for *Triticum urartu,* which is the species responsible for adding “A” genome in wheat*.* This may suggest the possible gene loss event in *Triticum urartu* leading to no “A” homoeolog of *ECA3* gene in wheat after polyploidization event. However, further advancements in wheat sequencing can clarify this fact.

### Effect of calcium stress on the phenotype of wheat

**Fig. 2:**
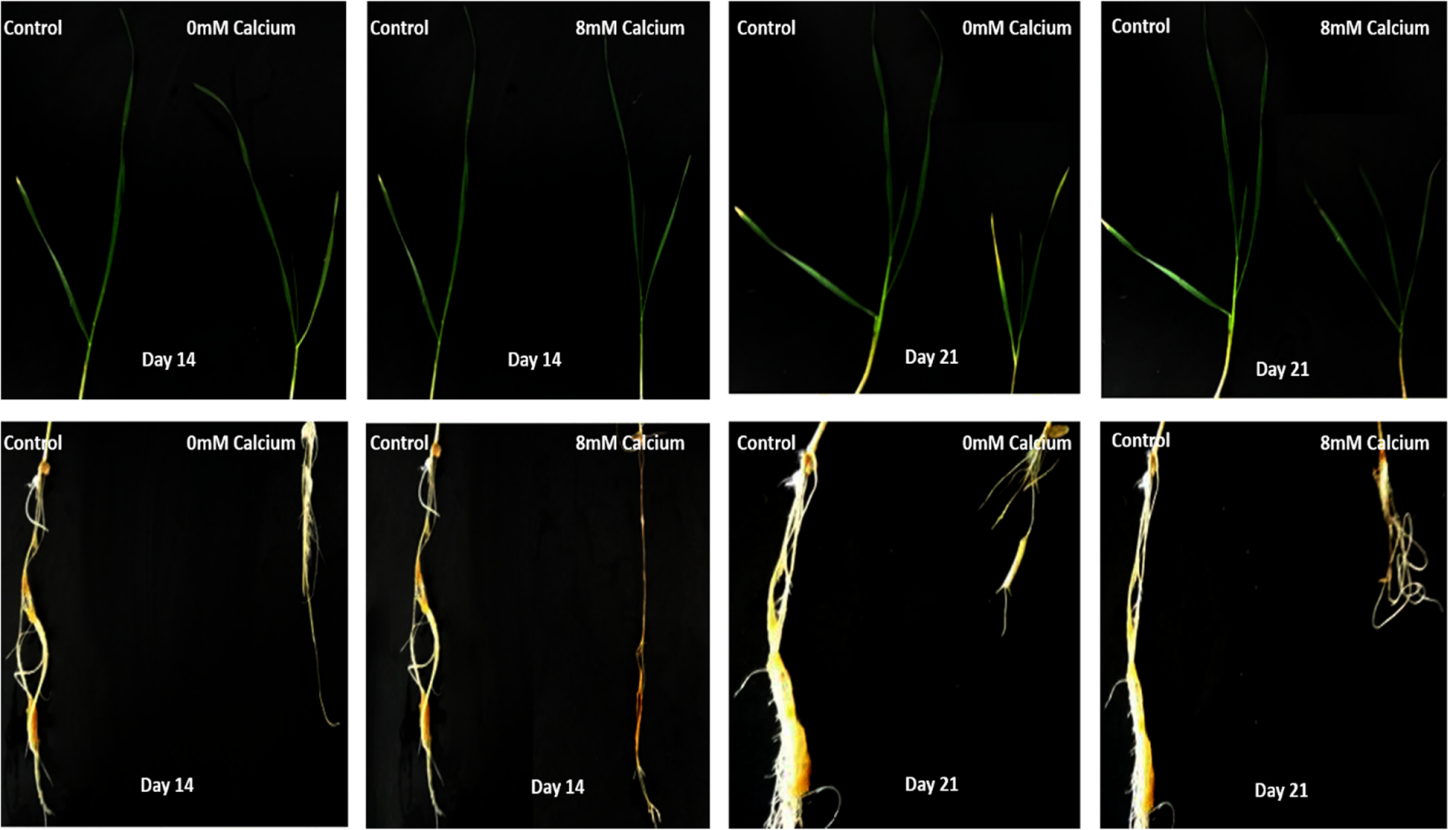
Growth of wheat plants using hydroponic culture on calcium deficiency, toxicity and control media on 14th and 21st day of growth. Reduction in volume of *Triticum aestivum* roots grown under calcium deficiency and toxicity as compared to control after 14 and 21 days of growth on control medium. The shoots grown under calcium deficiency and toxicity displayed chlorosis symptoms and reduction in length

**Fig. 3:**
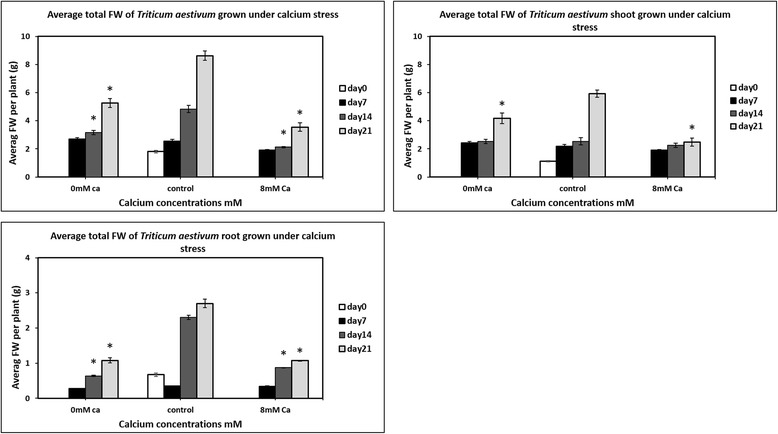
Average FW values of *Triticum aestivum* plants grown under calcium deficiency and toxicity plotted against average FW values of *Triticum aestivum* plants grown under control conditions. A significant difference was evaluated using Student’s t-test, where *P* < 0.05 * = significant difference. The data indicate that Plants grown under control conditions i.e., normal 2 mM Ca concentration in the solution grow well and gain more weight as compared to plants grown under calcium deficiency (0 mM Ca concentration in solution) and toxicity (8 mM ca concentration in solution)

Wheat plants were grown in hydroponics under calcium stress (both toxicity and deficiency) following fourteen days (referred as day 0) of growth on standard media. On day seven (i.e.) 7th day after transferring plants into deficiency and toxicity media, no symptoms of calcium deficiency and toxicity were noted. No significant difference in FW was measured at that time. The plants were allowed to grow for seven more days. On day fourteen symptoms of deficiency and toxicity were observed on plant roots (Fig. 2). The roots became narrow and thinner as compared to the control. However, no strong deficiency/toxicity symptoms were recorded on shoots. On the day twenty one, chlorosis of shoots was observed in plants grown under calcium deficiency and toxicity, as compared to plants grown under control condition (Fig. 2). There was a significant reduction in fresh weight (Fig. 3).

### Expression of Ca-ATPases under calcium stress

**Fig. 4:**
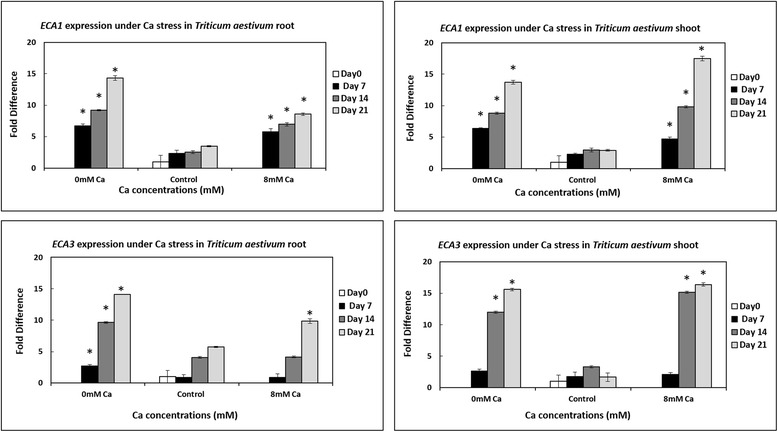
qRT-PCR data indicating the expression of *ECA1* and *ECA3* in *Triticum aestivum* shoots and roots under calcium deficiency/toxicity and control. The experiment was repeated thrice and three biological reps and three technical reps were used each time. The fold difference was evaluated relative to baseline D0 control. The significant differences in expression of *ECA1* and *ECA3* genes in plants grown under calcium deficiency and toxicity conditions as compared to plants grown under control conditions were evaluated using student’s t-test. Significant differences are indicated by * where *P* < 0.05. Standard error bars have been shown for data obtained from real time PCR. Y-axis shows the fold difference, whereas, the treatments are given on X-axis. Differences in colors of the bars are used to indicate the days of growth

**Fig. 5:**
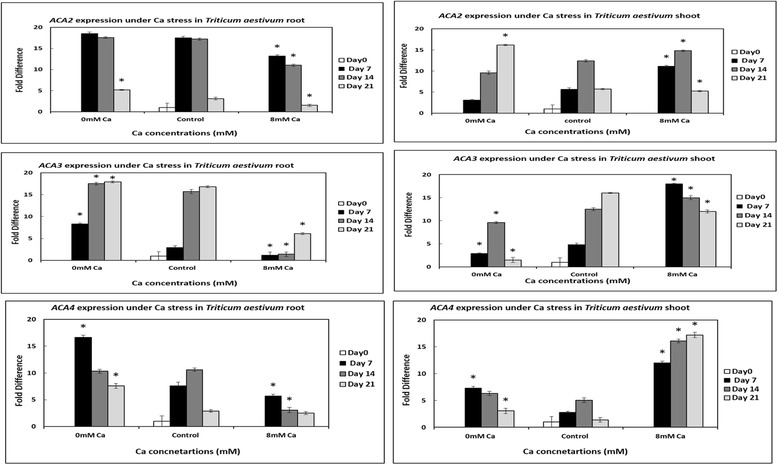
qRT-PCR data indicating the expression of *ACA2, ACA3 and ACA4* in *Triticum aestivum* shoots and roots under calcium deficiency/toxicity and control conditions. The experiment was repeated thrice and three biological reps and three technical reps were used each time. The fold difference was evaluated relative to baseline D0 control. The significant difference in expression of *ACA2*, *ACA3* and *ACA4* genes in plants grown under calcium stress (deficiency/toxicity) as compared to plants grown under control conditions was evaluated using student’s t-test. The significant differences are indicated by * where P < 0.05. Standard error bars have been shown for data obtained from real time PCR. Y-axis shows the fold difference, whereas, the treatments are given on X-axis. Differences in colors of the bars are used to indicate the days of growth

Three P_2B_- type (*ACA2*, *ACA3* and *ACA4*) and two P_2A_- type (*ECA1* and *ECA3*) calcium ATPases were chosen for gene expression analysis in *Triticum aestivum* grown under calcium stress using qRT-PCR. Expression profiling has shown that *ECA1* and *ECA3* are expressed in both roots and shoots of wheat plants when plants are grown under calcium deficiency and toxicity conditions (Fig. 4). Similarly, *ACA2* is expressed under calcium stress conditions in both roots and shoots (Fig. 4). However, expression of *ACA2* was observed to be more enhanced under calcium toxicity, as compared to deficiency. Moreover, *ACA3* and *ACA4* were expressed in both roots and shoots under calcium stress (Fig.5).

## Discussion

“Comparative genomics” has gained a lot of popularity in the present era, particularly in plant sciences. It provides an opportunity for the comparison of various genomic features such as DNA sequences, genes, and order of genes of different organisms. This type of study helps in the understanding of biological similarities and differences as well as the evolutionary relationships between organisms. Comparative genomics replaced the molecular marker technology with high throughput screening for “Crop improvement”. Through “Genome program”, key genes and their functions, can be identified which can be useful for crop improvement. For example, *Eutrema salsugineum* (formerly known as *Thellungiella halophila*), belongs to Brassicaceae, is native to eastern China’s saline soils and is widely used as a halophytic model for stress tolerance research in plants [36–38].The genome of this halophyte has been sequenced and published in 2013 [39]. The genome of several other species from this family, such as *Arabidopsis lyrata*, *Brassica rapa*, *Capsella rubella*, *Eutrema parvulum* ([40–43] has already been sequenced. This availability of whole-genome sequences of several species in *Brassicaceae* has opened a new era of comparative genomics for a better understanding of genome evolution of this plant family [43]. Similarly, Rice belongs to the family Poaceae and is closely related to other cereals such as maize, wheat, sugarcane, barley, sorghum and oats etc. There exist a high degree of conservation of phenotypic features across this family, synteny is conserved across the cereal genomes [44]. The availability of the genome sequence of rice synteny studies in cereals can be expanded from the macro scale reported to a more micro scale. Hence, rice can be very useful in “comparative genomics” for identifying other cereal genes. A similar approach was used in this study for the identification of P- type calcium ATPases in the newly sequenced wheat genome.

The rice database MSU [27] was used to retrieve *Oryza sativa* calcium ATPase sequences and were cross verified through another rice database RAP [29] and ARAMEMNON [28]. ARAMEMNON is a data source for plant membrane protein data and uses model plant *Arabidopsis thaliana* as a reference. The annotated rice calcium ATPase sequences were used to do BLAST searches in UniProtKB and Ensembl Plants [26] to retrieve other monocots calcium ATPases as given in Table 1. The retrieved sequences were used to construct a phylogenetic tree (Fig. 1) with MEGA version 7 using Maximum Likelihood method. The cladogram consists of two clades. One clade is composed of P_2A_- type ATPases (*ECA*s) and other clade is composed of P_2B_- type ATPases (*ACA*s). P_2B_ clade was further divided into ten main clades. Each clade was composed of one gene sequence from different species. This suggests the relatedness of calcium ATPases among different organisms, possibly indicating a common ancestor. The present analysis also revealed that there are nine different types of P_2B_ ATPases of wheat. *Brachypodium distachyon* and *Triticum urartu* also has the same number of P_2B_ -type ATPases. *Triticum urartu* adds “A” genome to modern hexaploid wheat. Whereas, *B. distachyon* is a wild grass whose genome has been sequenced recently [45]. It is proposed as a new model organism, for studying large genome grasses [46]. An earlier study done in 2008 based on micro collinearity between *Oryza sativa*, *Triticum aestivum* and *Brachypodium distachyon* has revealed that *Brachypodium distachyon* is more closely related to *Triticum aestivum* as compared to *Oryza sativa* [47]. In the present analysis, P_2_- type calcium ATPase sequences of *Triticum urartu* found to be closely related to *Triticum aestivum* P_2_- type calcium ATPases “A” homoeolog. It is because of the established fact that *Triticum urartu* adds “A” genome to the modern hexaploid wheat. The appearance of *Triticum urartu* and *Brachypodium distachyon* P_2_- type ATPases along with the *Triticum aestivum* homoeologs indicate the close genetic relationship between these two organisms. This finding further supports the suggestion that *Brachypodium distachyon* annotated genome can be quite useful in annotating wheat genome [48]. Similarly, *Oryza sativa* calcium ATPase sequences appeared closely related to *Sorghum bicolor* calcium ATPase sequences. Annotated *Oryza sativa* genome can be useful in annotating *Sorghum bicolor* genome.

The appearance of three clades of P_2A_- type ATPases is consistent with the previous findings that monocots have three P_2A_- type of ATPases as compared to *Arabidopsis thaliana* (a dicot) which possess four [2]. The clade of *ECA3* gene was composed of only two wheat homoeologs for this gene. The homoeolog “A” which is introduced by *Triticum urartu* was found to be completely missing. Interestingly, no *Triticum urartu ECA3* clade could be found in the tree. The databases were searched for *Triticum urartu ECA3* sequence but resulted in failure (Table 2). This observation may indicate two possibilities. Either the databases do not contain this sequences or those sequences are not annotated yet. The other possible reason might be that *ECA3* gene was under strong selection pressure in *Triticum urartu* during evolution. This result in “loss of” *ECA3* sequence in *Triticum urartu*. *Triticum urartu* adds “A” genome in the wheat. The two homoeologs of *ECA3* gene in wheat are 4BS and 4DS. As *ECA3* gene was lost in *Triticum urartu* as a result of “gene loss” event, no corresponding homoeolog could be spotted in *Triticum aestivum*. This information can be very useful for the further understanding of *Triticum aestivum* evolutionary history. However, experimental evidence is required to validate it as present study is based on the evidence available in the databases.

For expression profiling, *Triticum aestivum* plants were grown under calcium stress using hydroponic culture. Standard Lombnaes media [32] was used to grow plants for first fourteen days before transferring them to toxicity, deficiency and control media. The plants were kept under observation for calcium deficiency and toxicity symptoms after transferring them to deficient and toxic media. For first seven days of growth on calcium deficient and toxic media, no signs of deficiency and toxicity were observed. However, after further seven days, signs of calcium deficiency and toxicity began appearing on wheat roots. The clear symptom of calcium deficiency and toxicity on wheat shoots were noted only after 21 days of growth on calcium deficient and toxic media. Plants grown under calcium deficiency and toxicity were stunted as well as chlorotic (Fig. 2). Three plants were harvested after 14 days of growth on standard media and then at day7^th^, 14th and 21st of growth on calcium deficient and toxic media. The fresh weight was measured and student’s t-test was used to evaluate any significant differences (Fig. 3). The figure shows that after 7 days of growth on deficiency and toxicity media no significant difference occurred as compared to control in fresh weight values. The significant difference was observed after 14 days of growth and was also observed after 21 days. Similarly, in shoot FW only significant difference was observed on the 21st day. This shows that roots showed the more significant difference as compared to shoots and it can be observed on day 14th and 21st (Fig. 3). These results suggest that calcium stress has a more severe effect on *Triticum aestivum* roots as compared to shoots. The plants grown under stress have shorter and narrower roots as compared to plants grown under control. One possible reason may be the fact that roots are exposed directly to the deficiency/toxicity media (Fig. 3). Exposure of plant roots to the stress results in reducing root volume, hence overall surface area for absorption. This marks in lesser translocation of deficiency/toxicity media to the shoots leading to the lesser effect of deficiency/toxicity on them. The roots are at first place to get affected by the media changes, therefore, reduction in volume and length happened more in roots as compared to shoots. This results in the more significant difference in root fresh weight values as compared to plant shoots.

In the present study, it has been observed that P_2_- type ATPases are expressed in both roots and shoots of wheat plants under normal conditions within the cell as has been reported earlier [2, 49]. However, the expression of these genes gets enhanced when plants are exposed to calcium deficiency and toxicity (Fig. 4 and Fig. 5). This finding gives clues to the fact that likewise in dicots, monocots P_2_- type ATPases may also have possible roles in calcium ions homeostasis and calcium nutrition in cell. In fact, an increase in calcium levels within the cell can be responsible for the production of various toxic compounds which can bring damage to protein and nucleic acids as well as can disintegrate membrane lipids [50]. During toxicity (in present study), the increase in expression of P_2_ type- ATPases may have occurred to remove excess calcium from the cytosol to prevent over storage in cell organelles. This is consistent with the previous findings which suggest that P_2_- type calcium ATPases can cause the extrusion of Ca^2+^ ions from the cytosol and play role in the maintenance of low cytoplasmic Ca^2+^ions along with Ca^2+^/H^+^ exchanger-driven transporters [51]. The importance of P_2_- type calcium ATPases in calcium nutrition have also been established earlier. It has since long been known that P_2_- type calcium ATPases play role not only in uptake of Ca^2+^ ions but also in transport of these ions in root cells [52]. In the present study, the high expression of calcium ATPases during calcium deficiency in wheat roots and shoots suggest high activity of these proteins to get any available calcium in the medium or to transport the stored calcium from cell organelles to the cytosol.

Ca^2+^ ions (cytosol) transients have been observed under abiotic stresses in plants. It supports the belief that plants utilize Ca^2+^ions to generate a signaling pathway. This pathway possibly triggers the onset of events required as a defense response in plants [53, 54]. It is, therefore, very important for the cells to maintain low resting Ca^2+^ levels because of its role under stress conditions. Plants have evolved efficient mechanisms which keep the concentration of calcium at a constant level by exporting Ca^2+^ into the intracellular organelles or out of the cell [50]. Generally, the concentrations of free Ca^2+^ ions are in the range of 100–200 nM in the cytoplasm, 0.2–10 mM in the vacuole, ∼1 mM in the endoplasmic reticulum and 2–6 μM in chloroplast stroma [55]. Any fluctuations in these values are typically perceived as stress signals by plants. These elevations are further decoded by different proteins like CaM, CDPKs etc. which then generate stress specific physiological response [56].

Different plant proteins play role in maintaining homeostatic levels of calcium within cells under normal conditions by sequestering calcium ions to intracellular compartments. P_2_- type ATPases are believed to be among such proteins which are required to maintain low calcium cytosolic levels and are generally believed to have roles in abiotic stresses via calcium mediated signaling pathways. The expression of various P_2_- type ATPases is found to get upregulated under various abiotic stresses. For instance, it has been found that *ACA8* expression is upregulated in plants when they are exposed to cold stress [57]. The expression of *ACA2* and *ACA4* has been found to get enhanced under salt stress [17, 58]. Similarly, the up regulations in the expression of *ACA8* and *ACA9* in Arabidopsis seedlings under ABA (Abscisic acid) exposure further supports the belief that P_2_- type ATPases have possible roles in plants under abiotic stresses [59]. Likewise, the high expression of P_2_- type ATPases during calcium toxicity and deficiency conditions may also happened to trigger a signaling pathway to aware wheat plants about the surrounding calcium deficiency or calcium toxicity conditions. However, further experimental work based on cloning of genes and characterization using yeast models etc. is required to find out in details that how P_2A_- type ATPases are performing these activities during calcium stress in wheat plants.

## Conclusion

Overall, the study demonstrated that P_2_- type calcium ATPases are well conserved among different monocots. The genus *Brachypodium* seems to be very close to the genus *Triticum*. Hence, annotated *Brachypodium distachyon* genome can be quite useful to annotate *Triticum aestivum* genome. However, the genus *Sorghum* is more close to the genus *Oryza* as compared to other genus used in the study. Hence, annotated *Oryza sativa* genome can be very useful for the annotation of *Sorghum bicolor* genome. Furthermore, we purpose here that “loss of genes” may occur in original contributors of today’s hexaploid wheat resulting in loss of those “specific” genes in modern wheat. For example loss of *ECA3* gene in *Triticum urartu* resulted in no “A” homoeolog of this gene in today’s wheat. Additionally, we have found that P_2_- type calcium ATPases are expressed in both root and shoot under normal conditions in wheat plants. We have also found that P_2_- type ATPases in wheat are required during calcium toxicity to efflux excess Ca^2+^ ions out of the cytosol. Similarly, P_2_- type ATPases are also required for calcium uptake and transport. Furthermore, we have also found that P_2_- type ATPases might also have been involved in stress signaling in wheat.

## Abbreviations

ABA: Abscisic acid
*ACA*: *ACA*-auto inhibited calcium ATPases
CaM: Calmodulin
CDPK: Calmodulin like Domain Protein Kinase
CMBD: Calmodulin-binding Domains
ddH_2_0: Double Distilled Water
*ECA*: *ECA*s-endoplasmic reticulum calcium ATPases
FW: Fresh Weight
KCL: Potassium chloride
MEGA: Molecular Evolutionary Genetic Analysis
MSU: Michigan State University Rice Annotation Project
NaCl: Sodium chloride
PMCA: Plasma Membrane Calcium ATPase
RAP: The Rice Annotation Project
SERCA: Sarco-endoplasmic reticulum Ca^2+^pump
UniProtKB: The Universal Protein Resource Knowledgebase
